# Severe‐to‐Profound Hearing Loss and Mental Health: Initial Evidence That Cochlear Implantation Helps Alleviate Symptoms of Anxiety and Stress

**DOI:** 10.1111/coa.14326

**Published:** 2025-04-29

**Authors:** Paul McIlhiney, Osvaldo P. Almeida, Catherine M. Sucher, Robert H. Eikelboom, Dona M. P. Jayakody

**Affiliations:** ^1^ Ear Science Institute Australia Subiaco Western Australia Australia; ^2^ Department of Psychological Sciences The University of Western Australia Crawley Western Australia Australia; ^3^ UWA Medical School The University of Western Australia Crawley Western Australia Australia; ^4^ WA Centre for Health and Ageing The University of Western Australia Crawley Western Australia Australia; ^5^ Centre for Ear Sciences The University of Western Australia Crawley Western Australia Australia; ^6^ Curtin Medical School Curtin University Bentley Western Australia Australia; ^7^ Department of Speech Language Pathology and Audiology University of Pretoria Pretoria South Africa; ^8^ Curtin School of Allied Health Curtin University Bentley Western Australia Australia

**Keywords:** anxiety, cochlear implants, depression, hearing loss, longitudinal, stress

## Abstract

**Objectives:**

Mental‐health issues accounted for 418 million disability‐adjusted life years in 2019, costing the world economy approximately $5 trillion. Untreated hearing loss is a well‐known modifiable risk factor for mental‐health issues, with severe‐to‐profound hearing loss having the largest impact. Therefore, treatment of severe‐to‐profound hearing loss, namely with cochlear implantation, could help to alleviate psychological distress. However, previous studies have failed to include comprehensive measures of mental health or adequate controls. The current study thus aimed to conduct a controlled, longitudinal investigation of how cochlear implantation affects depression, anxiety and stress levels.

**Participants:**

Participants were 87 adults assigned to conditions based on hearing status: normal hearing (*n* = 44), received cochlear implant (*n* = 26) or untreated hearing loss (*n* = 17).

**Main Outcome Measures:**

The short‐form Depression Anxiety Stress Scale was given at four timepoints (baseline, 3 months, 6 months, 12 months). Data were analysed using linear mixed‐effects modelling.

**Results:**

Results showed that cochlear implants helped to stabilise anxiety and stress symptoms, while depression symptoms were observed to worsen over time despite treatment.

**Conclusion:**

Our findings suggest that treatment of severe‐to‐profound hearing loss with cochlear implantation was associated with a lessening of anxiety and stress scores, although the clinical significance of such changes remains uncertain. Due to the current study's non‐randomised treatment allocation, future randomised controlled trials are required for confirmation. The present findings help inform clinical and societal interventions for mental‐health issues associated with hearing loss.

**Summary**

- Severe‐to‐profound hearing loss can negatively impact mental health.
- We longitudinally assessed cochlear implants' influence on mental health.
- A cochlear‐implanted group was compared to untreated and normal controls with linear mixed‐effects analyses.
- Cochlear implantation was associated with lessening of anxiety and stress, but not depressive symptoms.
- The current findings encourage post‐implantation mental‐health care for cochlear‐implant recipients.

## 1 Introduction

In 2019, mental‐health issues accounted for 418 million disability‐adjusted life years and $5 trillion in economic losses [1], signalling the need for research on interventional measures. Untreated hearing loss (HL) has also been shown to be a modifiable risk factor of common mental‐health conditions [2, 3, 4], with more severe HL producing greater psychological distress [5]. Thus, treatment of severe‐to‐profound HL could provide mental‐health benefits for a significant proportion of the population. Currently, the gold‐standard treatment for moderately severe‐to‐profound HL is cochlear implantation, which involves auditory‐nerve stimulation using electrodes inserted directly into the cochlea. While cochlear implantation in adults improves quality of life [6], previous research investigating the effect of cochlear implantation on mental‐health outcomes has produced mixed results; only some studies show benefits, while others had methodological limitations [6, 7].

Although research on the quality of life of cochlear‐implant (CI) recipients has included mental‐health assessments, they were only brief and general [8]. Contrera et al. [8] used The Medical Outcome Study Short Form‐36, which includes 12 questions assessing general emotional problems in the previous 4 weeks; three ask whether emotional problems have affected work and productivity, while the rest ask about degrees of feeling energetic, calm, or down. Such quality‐of‐life measures provide a general indication of mental health, but do not finely assess the symptoms of depression, anxiety or stress. Studies that have more directly and comprehensively assessed the mental health of post‐operative CI recipients have focused mainly on depression and/or anxiety, and have produced mixed findings [9, 10, 11, 12, 13, 14, 15]; some studies found a beneficial effect [9, 10], while others did not [11, 12, 13, 14, 15]. Furthermore, only Castiglione et al. [10] included healthy and untreated control groups in their study.

Moreover, and to our knowledge, only two studies on CI‐recipient mental health have investigated depression, anxiety and stress concurrently [12, 14]. Results showed that cochlear implantation had negligible effects on depression, anxiety or stress scores over 12 months, though this may have been due to the low scores at study entry (i.e., floor effect). The findings of Knopke et al. [12], however, suggested that better CI‐recipient mental health was associated with better outcomes, with this effect being greater in those aged over 80 years than in those aged between 70 and 80 years. If the absence of control groups in the Knopke et al. studies is also considered, then it is clear that further longitudinal, controlled investigations of mental health in CI recipients are required. This need is further reinforced by the findings of a recent systematic review [6], which showed that several of the abovementioned studies used suboptimal statistical methods by failing to correct for multiple post hoc comparisons [12, 14, 15], potentially skewing results positively.

## 2 Objectives

The current study aimed to assess the longitudinal impact of cochlear implantation on depression, anxiety, and stress scores in middle‐aged‐to‐older adults with severe‐to‐profound HL. We hypothesised that CI recipients' depression, anxiety and stress scores would improve over a period of 12 months compared with untreated hearing‐impaired controls and would be comparable to controls without HL.

## 3 Methods

### 3.1 Study Design

This was an observational, longitudinal investigation of mental health in individuals with normal hearing (NH), cochlear‐implant (CI)‐assisted hearing and untreated moderately to severely impaired HL. Participants were assessed at baseline, 3, 6, and 12 months. This study was reviewed and approved by The University of Western Australia's Human Research Ethics Office (RA/4/1/7368), and reporting was based on STROBE guidelines.

### 3.2 Participants

**FIGURE 1:**
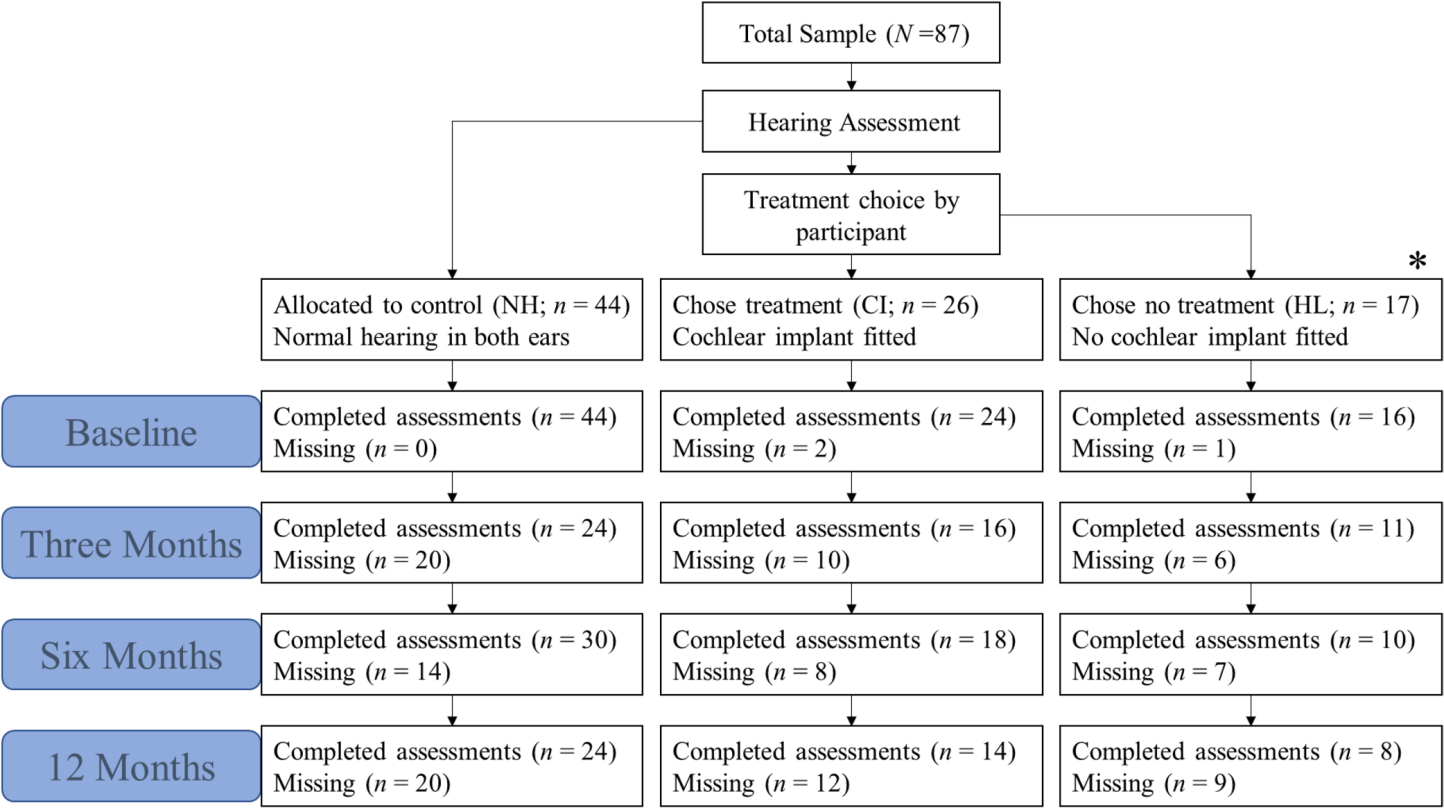
Flowchart depicting allocation of participants to normal‐hearing, cochlear‐implanted and untreated hearing‐loss groups, as well as sample sizes for each group at baseline, 3 months, 6 months and 12 months. *Note*: *HL‐group participants were offered and refused treatment with hearing aids; if they were deemed to be CI candidates, then they were excluded from the HL group.

Recruitment was via radio/newspaper advertisements, or the clinical services of the Ear Science Institute Australia Hearing Implant Centre. All participants in the NH group were recruited via advertisement, while those in the HL group were recruited via a mixture of advertisements and clinical services; all CI participants were recruited via the CI surgical waitlist of the hearing‐implant centre. To be included, participants had to be competent English speakers over 40 years old. Participants were excluded if they reported requiring assistance with activities of daily living, or had physical or psychological conditions that would prevent them from attending the scheduled assessments. In total, 87 men and women aged 41–89 years provided informed consent to participate (see Figure 1 below for more details).

### 3.3 Materials

#### 3.3.1 Hearing Assessment

A qualified audiologist conducted pure‐tone audiometric assessments (MIDIMATE 602 Audiometer, GN Otometrics Ltd., Sydney) in a sound‐proof booth. A better‐ear pure‐tone average at 0.5, 1, 2 and 4 kHz (BE4FAHL) was calculated. Participants were subsequently assigned to the NH group (BE4FAHL < 20 dB HL) or HL group (BE4FAHL ≥ 35 dB HL). Those in the CI group had received audiometric assessments previously and were recruited while on the waitlist for an implant (see Tables S1 and S2 for details about participants who received cochlear implants or received no treatment, respectively; https://osf.io/h3z2x/).

#### 3.3.2 Mental‐Health Assessment

The Depression Anxiety and Stress Scale (DASS‐21; [16]) was used to measure the severity and frequency of depression, anxiety and stress symptoms over the preceding 7 days. In the DASS‐21, depression, anxiety and stress are each measured with seven items. Each item presents a statement (e.g., ‘I felt that I was using a lot of nervous energy’), and agreement with the statement is rated on a four‐point Likert scale (i.e., *Never* [0], *Sometimes* [1], *Often* [2], *Almost Always* [3]). The seven Likert ratings for depression, anxiety, and stress were summed together and multiplied by two, giving three composite scores ranging from 0 to 42. Note that all CI recipients were implanted within 12 months of initial baseline measurements; due to this, three additional measurements were taken at 3, 6 and 12 months after the initial measurement, with the closest of these prior to implant date being used as the CI recipients' baseline measurement.

#### 3.3.3 Other Study Measures

Demographic information, age (in years), and sex (male/female) were also collected. Premorbid full‐scale IQ (FSIQ) was estimated using the *National Adult Reading Test* (NART; [17]).

#### 3.3.4 Statistical Analyses

Analyses were performed in RStudio (version 2023.6.0.421). Descriptive analyses were conducted first to check model assumptions. Linear mixed‐effects models were subsequently conducted using the *lme4*. Our mixed models included a single random effect of intercept, grouped by participant ID; the model also included age, sex and FSIQ as covariates to help control for baseline differences. To further account for the inclusion of individuals with asymmetrical or one‐sided HL, the absolute difference in 4FAHL between left and right ears was included as a covariate; baseline depression, anxiety and stress scores were also added to control for group differences in mental health at baseline. Effects of hearing status and timepoint (i.e., fixed effects) were assessed with ANOVA and follow‐up pairwise comparisons; no familywise error correction was used for pairwise comparisons, as each factor only comprised three comparison groups [18]. Following Johnson [19], model effect sizes were estimated with marginal *R* ^2^ (fixed‐effects only) and conditional *R* ^2^ (fixed + random effects). For succinctness, we have only reported significant effects in the results; full results can be found in Tables S4–S13 (https://osf.io/h3z2x/).

## 4 Results

### 4.1 Descriptive Statistics

There were significant differences between hearing‐status groups in age and FSIQ at baseline, supporting their inclusion as covariates in our models; baseline depression, anxiety and stress scores also showed significant differences, requiring their inclusion as covariates to ensure follow‐up differences were independent of baseline differences (Table 1). Despite non‐significant baseline differences, sex was still included to ensure against potential confounding effects. Normality of mental‐health estimates was acceptable ([20, 21]; Table S3). Residual normality and homoscedasticity were satisfied in our models (Figures S1–S6), though linear mixed‐effects models have shown robustness to residual non‐normality and heteroscedasticity [22]. Finally, although only 31 participants had data at all timepoints, more participant data were usable via the use of (restricted) maximum‐likelihood estimation in linear mixed models [23]. However, the total sample available for our covariate‐adjusted models was *N* = 69, as several participants had no follow‐up data (*n* = 14) or missing covariate data (*n* = 4).

**TABLE 1:** Demographics and clinical characteristics of sample at baseline.

		NH, *n* = 44	CI, *n* = 26	HL, *n* = 17	*p* ^a^
Age	Mean (SD)	57.48 (8.70)	66.04 (12.10)	68.18 (11.41)	**< 0.001**
	Range	41–75	41–86	41–89	
Sex	Male	14	13	6	0.343
	Female	30	13	11	
FSIQ	Mean (SD)	113.80 (6.10)	109.46 (7.31)	111.24 (7.34)	**0.044**
	Range	94–126	95–124	90–123	
Depression	Mean (SD)	3.41 (3.94)	6.58 (5.45)	8.25 (6.49)	**0.006**
	Range	0–20	0–18	0–24	
Anxiety	Mean (SD)	2.95 (2.81)	6.50 (6.11)	6.63 (6.10)	**0.008**
	Range	0–12	0–24	0–20	
Stress	Mean (SD)	7.00 (4.95)	12.58 (6.92)	13.75 (7.11)	**< 0.001**
	Range	0–16	0–30	2–28	
Smoking	Missing	9	3	4	N/A
	Never	27	10	10	
	Former	2	13	1	
	Second hand	2	0	2	
	Current	4	0	0	
Drinking	Missing	9	3	4	N/A
	Never	2	4	2	
	2–4/Month	15	5	5	
	2–3/Week	8	10	4	
	≥ 4/Week	10	4	2	
Exercise	Missing	9	3	4	0.250
	Yes	24	20	10	
	No	11	3	3	

*Note*: Bolded *p* values are significant.

Abbreviations: CI, cochlear implanted; FSIQ, full‐scale IQ; HL, untreated hearing loss; NH, normal hearing.

^a^ All *p* values were derived from Welch ANOVAs.

### 4.2 Linear Mixed‐Effects Modelling of Depression, Anxiety and Stress Measures

**FIGURE 2:**
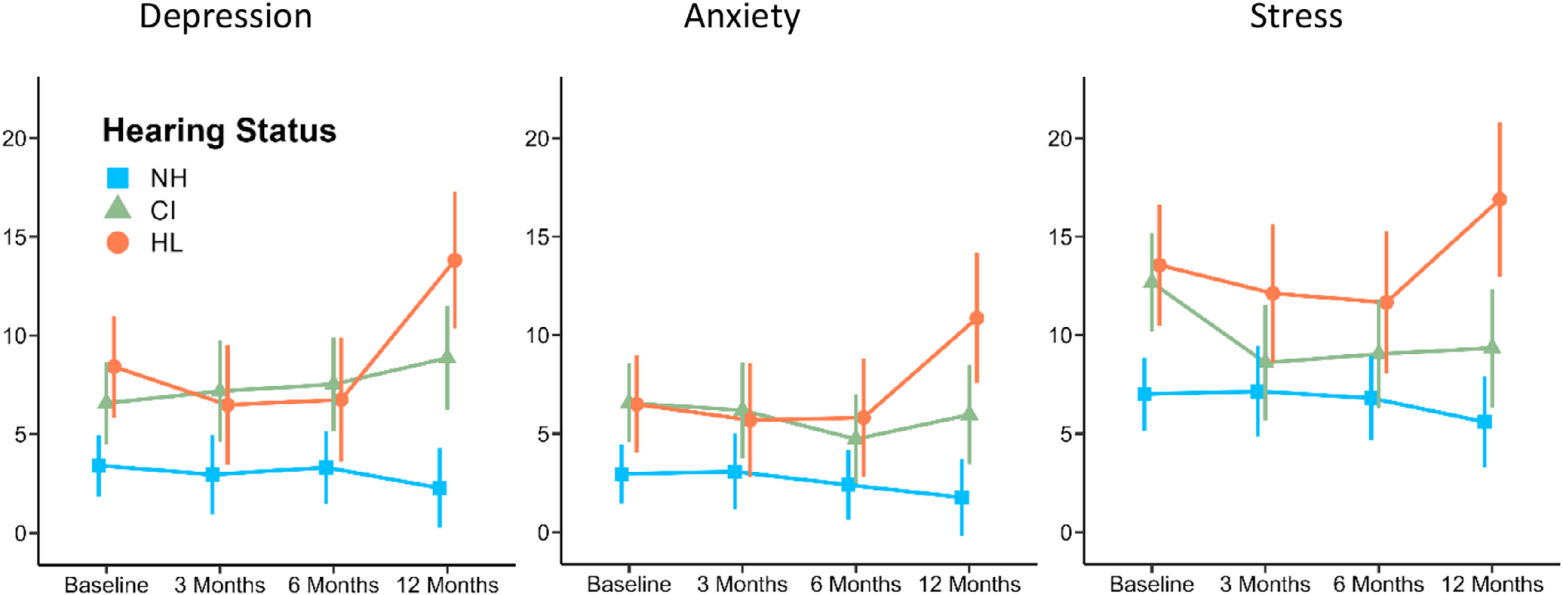
Line graphs depicting unadjusted estimated marginal means from linear mixed‐effects models of depression, anxiety and stress scores in NH, CI and HL groups across baseline, 3‐, 6 and 12‐month timepoints (*N* = 87). *Note*: Error bars depict 95% CIs. CI, cochlear implanted; HL, hearing loss; NH, normal hearing.

Before covariate adjustment, depression, anxiety and stress scores were generally similar in CI and HL groups, and numerically lower in the NH group (Figure 2); however, at 12 months follow‐up, CI‐group depression, anxiety and stress scores were significantly lower than those in the HL group (*p*_depression_ = 0.027, *p*_anxiety_ = 0.022, *p*_stress_ = 0.003), though only the stress scores were statistically similar to those in the NH group. The covariate‐adjusted linear mixed‐effects models (Table 2) demonstrated significant interactions between hearing status and time for depression (*F*[4, 83.04] = 4.04), anxiety (*F*[4, 67.64] = 2.84) and stress (*F*[4, 78.96] = 2.68); only the covariate of baseline scores was significant across all models (all *p*'s < 0.001), while the covariate of FSIQ was significant for anxiety and stress (all *p*'s ≤ 0.016). The marginal (and conditional) *R* ^2^ values for the depression, anxiety and stress models were 0.46 (0.54), 0.42 (0.60) and 0.44 (0.75), respectively.

**TABLE 2:** Effects of cochlear implants and time on depression, anxiety and stress scores, adjusted for age, sex, hearing‐loss asymmetry, FSIQ and baseline scores (*N* = 69).

		Adjusted mean scores after baseline [95% *CI*]			ANOVA *p* values		
Test	Months	NH	CI	HL	BS	WS	Int
Dep	3	3.98 [1.84, 6.12]	5.10 [1.93, 8.26]	4.39 [1.24, 7.54]	**0.045**	**0.001**	**0.005**
	6	4.22 [2.32, 6.12]	6.81 [4.00, 9.63]	4.38 [0.96, 7.79]			
	12	3.37 [1.26, 5.47]	9.15 [5.99, 12.31]	12.04 [8.44, 15.64]			
Anx	3	4.70 [2.79, 6.60]	3.04 [0.25, 5.84]	4.80 [2.03, 7.58]	0.088	**0.040**	**0.031**
	6	3.78 [2.06, 5.51]	3.52 [0.97, 6.06]	5.57 [2.54, 8.61]			
	12	3.51 [1.63, 5.38]	4.90 [2.07, 7.73]	10.21 [7.03, 13.39]			
Str	3	8.93 [6.42, 11.40]	6.69 [3.15, 10.20]	8.18 [4.30, 12.10]	0.545	0.447	**0.037**
	6	8.58 [6.21, 10.90]	8.11 [4.81, 10.40]	8.09 [3.97, 12.20]			
	12	7.58 [5.06, 10.10]	6.76 [3.19, 10.30]	12.93 [8.78, 17.10]			

*Note*: The bold values indicate statistical significance of *p* < 0.05.

Abbreviations: Anx, anxiety; BS, between subjects; CI, cochlear implanted; Dep, depression; HL, untreated hearing loss; Int, interaction; NH, normal hearing; Str, stress; WS, within subjects.

Follow‐up pairwise comparisons showed that both the CI and HL groups had significant increases from 3 to 12 months for depression scores (CI: *t* [102.40] = 2.11, *p* = 0.038, 95% *CI* [0.24, 7.86]; HL: *t* [83.58] = 3.67, *p* < 0.001, 95% *CI* [3.50, 11.80]); however, only the HL group showed an increase over the same period for anxiety (*t* [67.98] = 3.09, *p* = 0.003, 95% *CI* [1.92, 8.90]) and stress (*t* [73.62] = 2.52, *p* = 0.014, 95% *CI* [1.03, 8.83]) scores. Consequently, compared to the NH group at 12 months, depression scores were significantly higher in the CI (*t* [114.62] = 2.86, *p* = 0.005, 95% *CI* [1.77, 9.79]) and HL (*t* [123.97] = 4.08, *p* < 0.001, 95% *CI* [4.46, 12.88]) groups, while anxiety and stress scores were only higher in the HL group (*t*_anxiety_ [115.45] = 3.57, *p* = 0.001, 95% *CI* [2.98, 10.42]; *t*_stress_ [81.54] = 2.04, *p* = 0.045, 95% *CI* [0.12, 10.20]). Further, compared to the HL group at 12 months, the CI group had significantly lower anxiety scores (*t* [108.17] = 2.44, *p* = 0.017, 95% *CI* [0.99, 9.63]) and stress scores (*t* [84.55] = 2.28, *p* = 0.025, 95% *CI* [0.82, 12.06]), while CI‐ and NH‐group anxiety and stress scores were not statistically different (all *p*'s ≥ 0.583). Therefore, 12 months post‐operatively, CI recipients' depression scores were comparable to those with untreated, moderate‐to‐severe HL (i.e., higher and less stable), whereas CI recipients' anxiety and stress scores were comparable to those with NH (i.e., lower and more stable).

## 5 Discussion

Our aim was to assess the effect of cochlear implantation on the depression, anxiety and stress levels of adults and older adults with severe‐to‐profound HL. It was hypothesised that the mental health of cochlear‐implant recipients would improve over the 12‐month testing period, relative to controls. Our hypothesis and its associated predictions were only partially supported by the results.

Both the CI and HL groups showed some significant changes over time; namely, HL‐group depression, anxiety and stress scores increased over the 12‐month testing period, as did CI‐group depression scores. At 12 months, CI‐ and HL‐group depression scores were significantly higher than those in the NH group, as were HL‐group anxiety and stress scores. The HL group was generally observed to have the least stable mental health over the 12‐month testing period; however, CI recipients also demonstrated greater depressive symptoms following their treatment. Meanwhile, CI‐group anxiety and stress scores at 12 months were not significantly different from those in the NH group, and were significantly lower than those in the HL group. Therefore, while CI recipients experienced worsening of their mood, they were also observed to be less anxious and stressed than those with untreated HL.

The mixed results in our study add to a literature that has hitherto demonstrated inconsistent findings (for a review, see [6]). Specifically, some previous cochlear‐implant studies have found improvements in depression and anxiety symptoms over time [9, 10, 11, 15], or that lower depression and stress levels at 12 months post‐implant related to better cochlear‐implant‐related outcomes in older adults [12]. Conversely, some studies have found evidence of no improvement in depression and anxiety symptoms after implantation [11, 12, 13, 14]; note that while Claes et al. [11] found significant improvement after 6 months, depression and anxiety symptoms reverted to baseline levels at 12 months. Consequently, the current study's findings, and those of previous research, suggest substantial variability in the mental‐health outcomes of CI recipients.

This observed variability in mental‐health outcomes could relate to the well‐established variability in hearing‐related outcomes of cochlear‐implant recipients [24, 25, 26]. More specifically, cochlear implantation primarily aims to improve speech perception in recipients; however, while improvements are generally observed, they are variable and full functionality cannot currently be restored (for a review, see [27]). Furthermore, previous research has found a relationship between post‐implant mental health and subjective speech improvements [12, 28]. In an older‐adult sample, Knopke et al. [12] found that, at 12 months post‐implant, depression and anxiety levels correlated negatively with subjective speech perception and production (measured by the Nijmegen Cohclear Implant Questionnaire, NCIQ; [29]), while stress levels only did so with speech production. Similarly, at 12 months post‐implant, Völter et al. [28] demonstrated a negative correlation between depression levels and subjective speech perception (measured by the NCIQ) in older adults. Thus, there is evidence that those with better mental health experience better subjective speech ability following cochlear implantation; however, the direction of this relationship is not yet clear. Consequently, future research could also assess the relationship between objective speech improvements and mental health in cochlear‐implant recipients.

In terms of clinical implications, there are several issues to consider. First, mental‐health issues could inhibit cochlear‐implant recipients' engagement with their initial rehabilitation program; this could then lead to improper mapping of recipients' devices, as well as recipients receiving insufficient training to maximise their hearing‐related outcomes. It may then be advisable for mental‐health screening tests to become standard in implant‐candidacy test batteries and post‐implant care, thus allowing for issues to be detected and attended to earlier. This recommendation could be actioned by creating policy that mandates the use of formal mental‐health measures in pre‐ and post‐op care, along with the definition of referral pathways to mental‐health professionals (e.g., psychiatrists, psychologists, therapists, etc.) for any CI clients who may be experiencing mental‐health issues before and after their surgery. Furthermore, low mental‐health literacy has recently been found amongst hearing healthcare professionals [30, 31], meaning cochlear‐implant audiologists may not have sufficient competency or confidence to provide recommended mental‐health support. Mental‐health‐literacy training may then help cochlear‐implant audiologists to better support clients, namely by improving knowledge in counselling and referral pathways. Finally, unrealistic expectations of outcomes could play a role in mental‐health issues of cochlear‐implant recipients [32]; therefore, training audiologists to better manage expectations may help to improve the post‐implant mental health of some clients.

Regarding further limitations, treatment assignment was chosen by participants, rather than being randomised, meaning that it is unknown whether cochlear implantation affected mental health or vice versa. Moving forward, randomised controlled trials of the relationship between cochlear implantation and mental health are required. We also observed a significant degree of dropout across our timepoints; although we tried to alleviate this via (restricted) maximum‐likelihood estimation in our mixed‐model analyses, it is possible that this introduced bias if data were not missing at random. Restrictions from our inclusion and exclusion criteria may also have influenced our results. For instance, while restricting our sample to those aged 40 and over was intended to target those with a gradual, post‐lingual HL, it left the effects of pre‐lingual or early‐life–onset HL unknown; further, due to exclusion of those lacking functional independence from severe mental‐ or physical‐health conditions, our results cannot elucidate how these factors may impact the mental health of relevant CI recipients. The differing recruitment method between treatment and control groups may also be considered a limitation; namely, cochlear‐implant recipients were approached during assessment in a cochlear‐implant program, while control‐group participants either responded to radio/newspaper advertisements or were asked during routine audiological appointments. These differences in recruitment pool between groups could produce variance in willingness to participate across different demographics [33]. Furthermore, despite several participants having clinically significant symptomology, group means for our depression, anxiety and stress estimates were relatively low; therefore, potential improvement in our sample's mental health was limited. Moreover, the current study only followed participants for 12 months post‐implant, meaning mental health after 12 months was unknown; indeed, CI recipients could have experienced further mental health improvements after 12 months, or perhaps a worsening due to changes in their experience with their CIs (e.g., device failures, worse than expected outcomes). Future studies could therefore assess mental health up to 24 or 36 months after implantation, especially since the rehabilitation and adjustment period for cochlear implants can take 6 months or more. Our sample size, while larger than the typical cochlear‐implant study, was also limited, particularly in the untreated‐hearing‐loss and cochlear‐implant groups.

To conclude, the present results provide insight into the mental health of CI recipients, which may help to improve interventions and inform clinical practice in audiology.

## Author Contributions

**Paul McIlhiney:** writing – original draft, formal analysis, data curation, visualisation. **Osvaldo P. Almeida:** writing – review and editing, formal analysis. **Catherine M. Sucher:** writing – review and editing. **Robert H. Eikelboom:** writing – review and editing. **Dona M. P. Jayakody:** conceptualisation, methodology, supervision, project management, writing – review and editing.

## Ethics Statement

Ethics approval was granted by the University of Western Australia Human Research Ethics Committee (RA/4/1/7368).

## Conflicts of Interest

The authors declare no conflicts of interest.

## Peer Review

The peer review history for this article is available at https://www.webofscience.com/api/gateway/wos/peer‐review/10.1111/coa.14326.

## Supporting information


**Data S1.** Supporting Information.

## Data Availability

The data that support the findings of this study are openly available in OSF at https://osf.io/h3z2x/.
