# Supplementary material for: Severe‐to‐Profound Hearing Loss and Mental Health: Initial Evidence That Cochlear Implantation Helps Alleviate Symptoms of Anxiety and Stress
Source: Clin Otolaryngol. 2025 Apr 29;50(5):848–55. doi: 10.1111/coa.14326 (PMC12319481; doi:10.1111/coa.14326)
Supplement: Supplementary file 1 — Data S1. Supporting Information. [file COA-50-848-s001.docx]

**Online Supplement**

**Cochlear-Implant-Recipient Details**

| **Table S1**  *Hearing-Loss and Implant Details for Cochlear Implant Recipients (N = 26).* | | | |
| --- | --- | --- | --- |
|  |  | Frequency | Percentage |
| HL Aetiology | Age-Related | 5 | 19.23 |
|  | Hereditary | 3 | 11.54 |
|  | Meniere’s | 2 | 7.69 |
|  | Viral | 4 | 15.38 |
|  | Chronic OM | 2 | 7.69 |
|  | Otosclerosis | 1 | 3.85 |
|  | Head Injury | 1 | 3.85 |
|  | Noise Induced | 3 | 11.54 |
|  | Surgical Error | 1 | 3.85 |
|  | Sudden (Idiopathic) | 2 | 7.69 |
|  | Unknown | 2 | 7.69 |
| HL Onset Age | 60 – 79 | 5 | 19.23 |
|  | 40 – 59 | 7 | 26.92 |
|  | 20 – 39 | 9 | 34.62 |
|  | 0 – 19 | 5 | 19.23 |
| Previous HA Use | Yes | 18 | 69.23 |
|  | No | 2 | 7.69 |
|  | Unknown | 6 | 23.08 |
| Implant Laterality | Left | 17 | 65.38 |
|  | Right | 9 | 34.62 |
| Implant Model | CI24RE (CA)^a^ | 11 | 42.31 |
|  | CI24RE (ST)^a^ | 1 | 3.85 |
|  | CI512 (CA)^a^ | 5 | 19.23 |
|  | Concerto^b^ | 2 | 7.69 |
|  | Synchrony^b^ | 7 | 26.92 |
| Speech Processor | CP910^a^ | 16 | 61.54 |
|  | CP920^a^ | 1 | 3.85 |
|  | Sonnet^b^ | 5 | 19.23 |
|  | Rondo^b^ | 4 | 15.38 |
| Note: HL, Hearing Loss; OM, Otitis Media; HA, Hearing Aid; ^a^ Manufactured by Cochlear; ^b^ Manufactured by Med-El | | | |

**Untreated-Hearing-Loss Group Details**

| **Table S2**  *Hearing-Loss Details for Participants in the Untreated (No Hearing Aids) Hearing Loss Group (N = 17).* | | | |
| --- | --- | --- | --- |
|  |  | Frequency | Percentage |
| HL Aetiology | Age-Related | 17 | 100 |
| HL Age Noticed^a^ | 60 – 79 | 8 | 47.06 |
|  | 40 – 59 | 2 | 11.76 |
|  | 20 – 39 | 1 | 5.88 |
|  | 0 – 19 | 0 | 0 |
|  | Missing | 6 | 35.29 |
| HL Laterality | Left Worse | 0 | 0 |
|  | Right Worse | 1 | 5.88 |
|  | Both Similar^b^ | 16 | 94.12 |
| Note: HL, Hearing Loss; ^a^ Only had data from a demographic questionnaire that asked about when hearing loss was noticed instead of formal diagnosis; ^b^ Judged to be similar if difference between ears was within 10dB. | | | |

**Full Descriptive Statistics**

| **Table S3**  *Full Descriptive Statistics for Depression, Anxiety, and Stress Scores at Baseline, Three, Six, and 12 Months (N = 87).* | | | | | | | | | | | | | |
| --- | --- | --- | --- | --- | --- | --- | --- | --- | --- | --- | --- | --- | --- |
|  |  | Depression | | | | Anxiety | | | | Stress | | | |
|  |  | T1 | T2 | T3 | T4 | T1 | T2 | T3 | T4 | T1 | T2 | T3 | T4 |
| NH | *n* | 44 | 24 | 30 | 24 | 44 | 24 | 30 | 24 | 44 | 24 | 30 | 24 |
|  | Miss | 0 | 20 | 14 | 20 | 0 | 20 | 14 | 20 | 0 | 20 | 14 | 20 |
|  | Mean | 3.41 | 2.92 | 3.47 | 2.00 | 2.95 | 3.08 | 2.50 | 1.50 | 7.00 | 7.33 | 6.73 | 4.83 |
|  | *SD* | 3.94 | 3.12 | 4.30 | 2.21 | 2.81 | 3.12 | 3.20 | 2.30 | 4.95 | 4.78 | 6.23 | 5.00 |
|  | Range | 0 – 20 | 0 – 10 | 0 – 16 | 0 – 8 | 0 – 12 | 0 – 10 | 0 – 12 | 0 – 8 | 0 – 16 | 0 – 16 | 0 – 24 | 0 – 16 |
|  | Skew | **2.11** | .95 | 1.62 | 1.06 | 1.13 | .93 | 1.83 | 2.03 | .09 | .08 | 1.15 | .99 |
|  | Kurt | 6.34 | -.28 | 2.19 | .83 | 1.26 | .17 | 3.55 | 3.94 | -1.17 | -1.00 | .98 | -.08 |
| CI | *n* | 24 | 15 | 18 | 14 | 24 | 15 | 18 | 14 | 24 | 15 | 18 | 14 |
|  | Miss | 2 | 11 | 8 | 12 | 2 | 11 | 8 | 12 | 2 | 11 | 8 | 12 |
|  | Mean | 6.58 | 7.07 | 7.56 | 8.43 | 6.50 | 5.20 | 5.11 | 5.86 | 12.58 | 7.87 | 9.22 | 9.00 |
|  | *SD* | 5.45 | 6.92 | 5.51 | 7.97 | 6.11 | 7.81 | 4.66 | 7.17 | 6.92 | 6.52 | 6.62 | 5.86 |
|  | Range | 0 – 18 | 0 – 24 | 0 – 24 | 0 – 24 | 0 – 24 | 0 – 28 | 0 – 16 | 0 – 24 | 0 – 30 | 0 – 22 | 0 – 22 | 0 – 20 |
|  | Skew | .54 | 1.33 | 1.44 | .72 | 1.24 | **2.14** | .77 | 1.68 | .31 | .80 | .27 | .26 |
|  | Kurt | -.68 | 1.31 | 3.91 | -.66 | 1.61 | 4.68 | -.02 | 2.41 | .68 | -.24 | -.72 | -.12 |
| HL | *n* | 16 | 11 | 10 | 8 | 16 | 11 | 10 | 8 | 16 | 11 | 10 | 8 |
|  | Miss | 1 | 6 | 7 | 9 | 1 | 6 | 7 | 9 | 1 | 6 | 7 | 9 |
|  | Mean | 8.25 | 6.36 | 6.40 | 12.75 | 6.63 | 6.00 | 5.60 | 9.75 | 13.75 | 12.55 | 12.00 | 16.00 |
|  | *SD* | 6.49 | 5.05 | 4.60 | 11.00 | 6.10 | 6.63 | 5.64 | 9.47 | 7.11 | 6.20 | 9.57 | 10.58 |
|  | Range | 0 – 24 | 0 – 18 | 0 – 14 | 0 – 30 | 0 – 20 | 0 – 24 | 0 – 18 | 0 – 28 | 2 – 28 | 2 – 22 | 0 – 32 | 2 – 32 |
|  | Skew | 1.25 | 1.26 | .18 | .55 | .77 | **2.21** | 1.33 | .97 | .38 | -.19 | 1.16 | -.06 |
|  | Kurt | 1.37 | 1.99 | -1.25 | -1.10 | -.33 | 6.07 | 1.62 | .74 | .13 | -.75 | 1.02 | -.72 |
| *Note*: NH, Normal Hearing; CI, Cochlear Implanted; HL, Hearing Loss; T1, Baseline; T2, Three Months; T3, Six Months; T4, 12 Months; Miss, Missing; Skew, Skewness; Kurt, Kurtosis; Bolded skewness and kurtosis values exceeded our criteria for normality (Schmider et al., 2010; West et al., 1995). | | | | | | | | | | | | | |

***Residual-Normality Plots***

**Figure S1**

*Residual Normality Plot for Depression Mixed Model.*


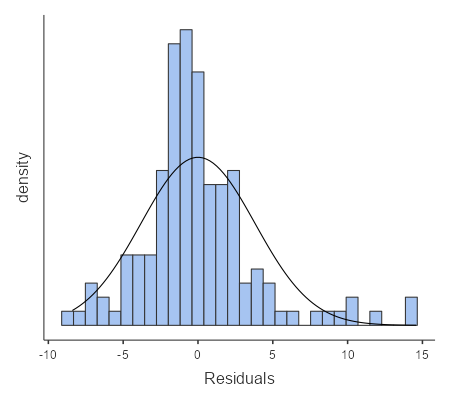


**Figure S2**

*Residual Normality Plot for Anxiety Mixed Model.*


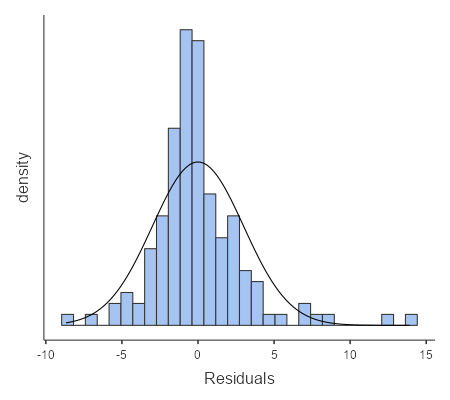


**Figure S3**

*Residual Normality Plot for Stress Mixed Model.*


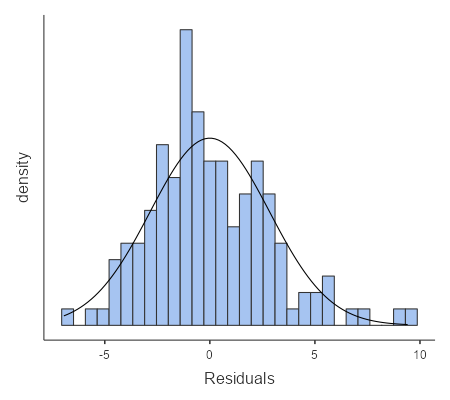


***Residuals-to-Predicted Plots***

**Figure S4**

*Residual-to-Predicted Plot for Depression Mixed Model.*


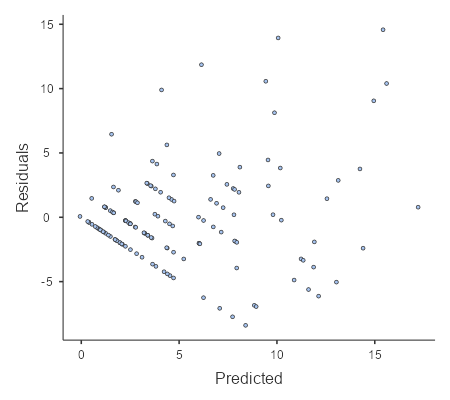


**Figure S5**

*Residual-to-Predicted Plot for Anxiety Mixed Model.*


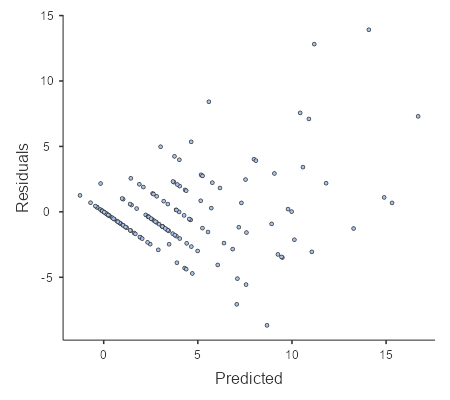


**Figure S6**

*Residual-to-Predicted Plot for Stress Mixed Model.*


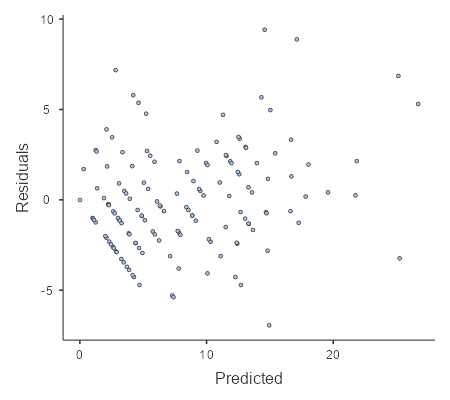


**Full Linear Mixed-Effects Modelling Output**

| **Table S4**  *Fixed Effects, Random Effects, and Model Statistics for Linear Mixed-Effects Model of Depression Scores.* | | | | | | |
| --- | --- | --- | --- | --- | --- | --- |
| Predictors | Estimates | *SE* | df | *t* | *p* | 95% *CI* |
| Intercept | 10.70 | 8.30 | 47.48 | 1.29 | .204 | -5.99, 27.39 |
| CI | 1.12 | 2.06 | 116.59 | 0.55 | .586 | -2.95, 5.19 |
| HL | 0.41 | 1.95 | 117.04 | 0.21 | .834 | -3.46, 4.28 |
| Six Months | 0.24 | 1.21 | 73.44 | 0.20 | .842 | -2.16, 2.65 |
| 12 Months | -0.61 | 1.27 | 70.57 | -0.48 | .633 | -3.15, 1.93 |
| Baseline Scores | 0.52 | 0.10 | 60.18 | 5.08 | **<.001** | 0.32, 0.73 |
| HL Asymmetry | -0.00 | 0.03 | 48.01 | -0.06 | .953 | -0.07, 0.07 |
| Age | 0.00 | 0.05 | 45.06 | 0.02 | .985 | -0.10, 0.10 |
| Sex | -0.42 | 1.04 | 46.01 | -0.40 | .689 | -2.50, 1.67 |
| FSIQ | -0.08 | 0.07 | 47.31 | -1.07 | .289 | -0.22, 0.07 |
| CI*Six Months | 1.47 | 2.15 | 90.93 | 0.69 | .495 | -2.79, 5.74 |
| HL*Six Months | -0.26 | 2.34 | 77.86 | -0.11 | .913 | -4.91, 4.40 |
| CI*12 Months | 4.66 | 2.30 | 92.11 | 2.02 | **.046** | 0.08, 9.24 |
| HL*12 Months | 8.26 | 2.44 | 80.08 | 3.38 | **.001** | 3.40, 13.12 |
| Random Effects | | | | | | |
| σ^2^ | 18.53 | | | | | |
| τ_00_ _ID_ | 4.56 | | | | | |
| *ICC* | 0.20 | | | | | |
| *N* _ID_ | 69 | | | | | |
| Model Statistics | | | | | | |
| Observations | 144 | | | | | |
| Marginal R^2^ | 0.375 | | | | | |
| Conditional R^2^ | 0.498 | | | | | |
| AIC | 868.065 | | | | | |
| Log-Likelihood | -418.033 | | | | | |

*Note*: Bolded *p* values are statistically significant; NH, Normal Hearing; CI, Cochlear Implanted; HL, Unaided Hearing Loss.

| **Table S5**  *Fixed Effects, Random Effects, and Model Statistics for Linear Mixed-Effects Model of Anxiety Scores.* | | | | | | |
| --- | --- | --- | --- | --- | --- | --- |
| Predictors | Estimates | *SE* | df | *t* | *p* | 95% *CI* |
| Intercept | 29.12 | 7.79 | 38.12 | 3.74 | **.001** | 13.35, 44.88 |
| CI | -1.65 | 1.81 | 101.66 | -0.91 | .363 | -5.24, 1.93 |
| HL | 0.10 | 1.72 | 99.07 | 0.06 | .953 | -3.30, 3.51 |
| Six Months | -0.91 | 1.01 | 58.57 | -0.91 | .368 | -2.93, 1.10 |
| 12 Months | -1.19 | 1.06 | 55.47 | -1.13 | .265 | -3.32, 0.93 |
| Baseline Scores | 0.48 | 0.10 | 40.42 | 4.87 | **<.001** | 0.28, 0.67 |
| HL Asymmetry | 0.02 | 0.03 | 37.43 | 0.65 | .521 | -0.04, 0.08 |
| Age | -0.01 | 0.05 | 36.30 | -0.24 | .810 | -0.11, 0.08 |
| Sex | -1.47 | 1.01 | 34.41 | -1.46 | .153 | -3.51, 0.57 |
| FSIQ | -0.21 | 0.07 | 37.14 | -3.15 | **.003** | -0.35, -0.08 |
| CI*Six Months | 1.39 | 1.82 | 76.93 | 0.76 | .448 | -2.23, 5.00 |
| HL*Six Months | 1.69 | 1.95 | 62.80 | 0.86 | .392 | -2.22, 5.59 |
| CI*12 Months | 3.05 | 1.95 | 77.99 | 1.57 | .121 | -0.82, 6.92 |
| HL*12 Months | 6.60 | 2.04 | 64.43 | 3.23 | **.002** | 2.52, 10.68 |
| Random Effects | | | | | | |
| σ^2^ | 12.65 | | | | | |
| τ_00_ _ID_ | 5.91 | | | | | |
| *ICC* | 0.32 | | | | | |
| *N* _ID_ | 69 | | | | | |
| Model Statistics | | | | | | |
| Observations | 144 | | | | | |
| Marginal R^2^ | 0.385 | | | | | |
| Conditional R^2^ | 0.581 | | | | | |
| AIC | 833.761 | | | | | |
| Log-Likelihood | -400.881 | | | | | |

*Note*: Bolded *p* values are statistically significant; NH, Normal Hearing; CI, Cochlear Implanted; HL, Unaided Hearing Loss.

| **Table S6**  *Fixed Effects, Random Effects, and Model Statistics for Linear Mixed-Effects Model of Stress Scores.* | | | | | | |
| --- | --- | --- | --- | --- | --- | --- |
| Predictors | Estimates | *SE* | df | *t* | *p* | 95% *CI* |
| Intercept | 29.06 | 10.85 | 57.65 | 2.68 | **.010** | 7.33, 50.79 |
| CI | -2.25 | 2.34 | 95.41 | -0.96 | .340 | -6.89, 2.40 |
| HL | -0.75 | 2.49 | 86.98 | -0.30 | .764 | -5.70, 4.19 |
| Six Months | -0.36 | 1.06 | 72.78 | -0.34 | .735 | -2.46, 1.75 |
| 12 Months | -1.36 | 1.11 | 69.90 | -1.23 | .224 | -3.56, 0.85 |
| Baseline Scores | 0.65 | 0.12 | 58.11 | 5.42 | **<.001** | 0.41, 0.89 |
| HL Asymmetry | -0.05 | 0.05 | 57.12 | -1.10 | .275 | -0.14, 0.04 |
| Age | 0.03 | 0.07 | 57.51 | 0.41 | .680 | -0.11, 0.17 |
| Sex | -0.32 | 1.41 | 54.79 | -0.23 | .823 | -3.14, 2.51 |
| FSIQ | -0.24 | 0.10 | 56.72 | -2.49 | **.016** | -0.43, -0.05 |
| CI*Six Months | 1.78 | 1.96 | 86.66 | 0.91 | .366 | -2.11, 5.67 |
| HL*Six Months | 0.26 | 2.06 | 75.38 | 0.13 | .899 | -3.84, 4.36 |
| CI*12 Months | 1.43 | 2.10 | 86.47 | 0.68 | .497 | -2.74, 5.60 |
| HL*12 Months | 6.10 | 2.16 | 76.21 | 2.83 | **.006** | 1.81, 10.40 |
| Random Effects | | | | | | |
| σ^2^ | 13.27 | | | | | |
| τ_00_ _ID_ | 17.75 | | | | | |
| *ICC* | 0.57 | | | | | |
| *N* _ID_ | 69 | | | | | |
| Model Statistics | | | | | | |
| Observations | 144 | | | | | |
| Marginal R^2^ | 0.401 | | | | | |
| Conditional R^2^ | 0.744 | | | | | |
| AIC | 877.865 | | | | | |
| Log-Likelihood | -422.932 | | | | | |

*Note*: Bolded *p* values are statistically significant; NH, Normal Hearing; CI, Cochlear Implanted; HL, Unaided Hearing Loss.

| **Table S7**  *Results of ANOVAs for Linear Mixed-Effects Models of Depression, Anxiety, and Stress Scores (N = 69).* | | | | |
| --- | --- | --- | --- | --- |
|  | Variable | *F* | *df*, *df*_error_ | *p* |
| Depression | Hear Stat | 3.30 | 2, 50.45 | **.045** |
|  | Time | 7.06 | 2, 84.16 | **.001** |
|  | Hear Stat*Time | 4.04 | 4, 83.04 | **.005** |
|  | Baseline | 25.80 | 1, 60.18 | **<.001** |
|  | Hear Asym | 0.00 | 1, 48.01 | .953 |
|  | Age | 0.00 | 1, 45.06 | .985 |
|  | Sex | 0.16 | 1, 46.01 | .689 |
|  | FSIQ | 1.15 | 1, 47.31 | .289 |
| Anxiety | Hear Stat | 2.59 | 2, 38.73 | .088 |
|  | Time | 3.38 | 2, 69.23 | **.040** |
|  | Hear Stat*Time | 2.84 | 4, 67.64 | **.031** |
|  | Baseline | 23.76 | 1, 40.42 | **<.001** |
|  | Hear Asym | 0.42 | 1, 37.43 | .521 |
|  | Age | 0.06 | 1, 36.30 | .810 |
|  | Sex | 2.14 | 1, 34.41 | .153 |
|  | FSIQ | 9.91 | 1, 37.14 | **.003** |
| Stress | Hear Stat | 0.61 | 2, 58.23 | .545 |
|  | Time | 0.81 | 2, 79.70 | .447 |
|  | Hear Stat*Time | 2.68 | 4, 78.96 | **.037** |
|  | Baseline | 29.34 | 1, 58.11 | **<.001** |
|  | Hear Asym | 1.21 | 1, 57.12 | .275 |
|  | Age | 0.17 | 1, 57.51 | .680 |
|  | Sex | 0.05 | 1, 54.79 | .823 |
|  | FSIQ | 6.22 | 1, 56.72 | **.016** |

*Note*: Bolded p values are statistically significant; Hear Stat, Hearing Status; Hear Asym, Hearing Asymmetry; FSIQ, Full-Scale Intelligence Quotient.

| **Table S8**  *Pairwise Comparisons of Differences in Depression EMMs between Hearing-Status Groups at Three, Six, and 12 Months (N = 69).* | | | | | | |
| --- | --- | --- | --- | --- | --- | --- |
| Timepoint | Comparison | *t* | *df* | *p* | 95% *CI* | |
|  |  |  |  |  | Lower | Upper |
| Three Months | NH – CI | -0.55 | 116.59 | .586 | -5.19 | 2.95 |
|  | NH – HL | -0.21 | 117.04 | .834 | -4.28 | 3.46 |
|  | CI – HL | 0.31 | 112.56 | .758 | -3.84 | 5.27 |
| Six Months | NH – CI | -1.44 | 101.08 | .152 | -6.16 | 0.97 |
|  | NH – HL | -0.08 | 117.65 | .938 | -4.12 | 3.81 |
|  | CI – HL | 1.09 | 110.33 | .279 | -2.00 | 6.88 |
| 12 Months | NH – CI | -2.86 | 114.62 | **.005** | -9.79 | -1.77 |
|  | NH – HL | -4.08 | 123.97 | **<.001** | -12.88 | -4.46 |
|  | CI – HL | -1.18 | 118.98 | .239 | -7.72 | 1.94 |

*Note*: Bolded *p* values are statistically significant; EMM, Estimated Marginal Mean; NH, Normal Hearing; CI, Cochlear Implanted; HL, Untreated Hearing Loss.

| **Table S9**  *Pairwise Comparisons of Differences in Depression EMMs between Timepoints in NH, HA, and HL Groups (N = 69).* | | | | | | |
| --- | --- | --- | --- | --- | --- | --- |
| Group | Comparison | *t* | *df* | *p* | 95% *CI* | |
|  |  |  |  |  | Lower | Upper |
| NH | Three Months – Six Months | -0.20 | 73.44 | .842 | -2.65 | 2.16 |
|  | Three Months – 12 Months | 0.48 | 70.57 | .633 | -1.93 | 3.15 |
|  | Six Months – 12 Months | 0.71 | 70.01 | .483 | -1.56 | 3.26 |
| CI | Three Months – Six Months | -0.97 | 100.34 | .334 | -5.22 | 1.79 |
|  | Three Months – 12 Months | -2.11 | 102.40 | **.038** | -7.86 | -0.24 |
|  | Six Months – 12 Months | -1.32 | 73.74 | .190 | -5.84 | 1.18 |
| HL | Three Months – Six Months | 0.01 | 79.50 | .995 | -3.96 | 3.99 |
|  | Three Months – 12 Months | -3.67 | 83.58 | **<.001** | -11.80 | -3.50 |
|  | Six Months – 12 Months | -3.52 | 87.42 | **.001** | -11.98 | -3.34 |

*Note*: Bolded *p* values are statistically significant; EMM, Estimated Marginal Mean; NH, Normal Hearing; CI, Cochlear Implanted; HL, Unaided Hearing Loss.

| **Table S10**  *Pairwise Comparisons of Differences in Anxiety EMMs between Hearing-Status Groups at Three, Six, and 12 Months (N = 69).* | | | | | | |
| --- | --- | --- | --- | --- | --- | --- |
| Timepoint | Comparison | *t* | *df* | *p* | 95% *CI* | |
|  |  |  |  |  | Lower | Upper |
| Three Months | NH – CI | 0.91 | 101.66 | .363 | -1.93 | 5.24 |
|  | NH – HL | -0.06 | 99.07 | .953 | -3.51 | 3.30 |
|  | CI – HL | -0.85 | 97.23 | .396 | -5.85 | 2.34 |
| Six Months | NH – CI | 0.16 | 81.24 | .870 | -2.98 | 3.51 |
|  | NH – HL | -1.00 | 104.72 | .319 | -5.32 | 1.75 |
|  | CI – HL | -1.02 | 94.37 | .311 | -6.06 | 1.95 |
| 12 Months | NH – CI | -0.77 | 101.35 | .443 | -4.99 | 2.20 |
|  | NH – HL | -3.57 | 115.45 | **.001** | -10.42 | -2.98 |
|  | CI – HL | -2.44 | 108.17 | **.017** | -9.63 | -0.99 |

*Note*: Bolded *p* values are statistically significant; EMM, Estimated Marginal Mean; NH, Normal Hearing; CI, Cochlear Implanted; HL, Untreated Hearing Loss.

| **Table S11**  *Pairwise Comparisons of Differences in Anxiety EMMs between Timepoints in NH, HA, and HL Groups (N = 69).* | | | | | | |
| --- | --- | --- | --- | --- | --- | --- |
| Group | Comparison | *t* | *df* | *p* | 95% *CI* | |
|  |  |  |  |  | Lower | Upper |
| NH | Three Months – Six Months | 0.91 | 58.57 | .368 | -1.10 | 2.93 |
|  | Three Months – 12 Months | 1.13 | 55.47 | .265 | -0.93 | 3.32 |
|  | Six Months – 12 Months | 0.28 | 55.41 | .783 | -1.74 | 2.30 |
| CI | Three Months – Six Months | -0.31 | 87.45 | .755 | -3.47 | 2.52 |
|  | Three Months – 12 Months | -1.14 | 90.35 | .257 | -5.09 | 1.38 |
|  | Six Months – 12 Months | -0.94 | 58.60 | .351 | -4.33 | 1.56 |
| HL | Three Months – Six Months | -0.46 | 64.33 | .646 | -4.11 | 2.57 |
|  | Three Months – 12 Months | -3.09 | 67.98 | **.003** | -8.90 | -1.92 |
|  | Six Months – 12 Months | -2.53 | 70.48 | **.014** | -8.29 | -0.98 |

*Note*: Bolded *p* values are statistically significant; EMM, Estimated Marginal Mean; NH, Normal Hearing; CI, Cochlear Implanted; HL, Unaided Hearing Loss.

| **Table S12**  *Pairwise Comparisons of Differences in Stress EMMs between Hearing-Status Groups at Three, Six, and 12 Months (N = 69).* | | | | | | |
| --- | --- | --- | --- | --- | --- | --- |
| Timepoint | Comparison | *t* | *df* | *p* | 95% *CI* | |
|  |  |  |  |  | Lower | Upper |
| Three Months | NH – CI | 1.19 | 92.88 | .238 | -1.92 | 7.63 |
|  | NH – HL | 0.43 | 85.83 | .669 | -4.05 | 6.29 |
|  | CI – HL | -0.59 | 91.18 | .558 | -7.62 | 4.14 |
| Six Months | NH – CI | 0.51 | 73.34 | .612 | -3.21 | 5.42 |
|  | NH – HL | 0.46 | 88.98 | .649 | -4.04 | 6.45 |
|  | CI – HL | 0.04 | 82.10 | .971 | -5.43 | 5.63 |
| 12 Months | NH – CI | 0.55 | 86.09 | .583 | -3.33 | 5.89 |
|  | NH – HL | -2.04 | 81.54 | **.045** | -10.20 | -0.12 |
|  | CI – HL | -2.28 | 84.55 | **.025** | -12.06 | -0.82 |

*Note*: Bolded *p* values are statistically significant; EMM, Estimated Marginal Mean; NH, Normal Hearing; CI, Cochlear Implanted; HL, Untreated Hearing Loss.

| **Table S13**  *Pairwise Comparisons of Differences in Stress EMMs between Timepoints in NH, HA, and HL Groups (N = 69).* | | | | | | |
| --- | --- | --- | --- | --- | --- | --- |
| Group | Comparison | *t* | *df* | *p* | 95% *CI* | |
|  |  |  |  |  | Lower | Upper |
| NH | Three Months – Six Months | 0.36 | 69.24 | .723 | -1.81 | 2.60 |
|  | Three Months – 12 Months | 1.19 | 67.47 | .238 | -0.91 | 3.61 |
|  | Six Months – 12 Months | 0.90 | 69.33 | .373 | -1.17 | 3.08 |
| CI | Three Months – Six Months | -0.81 | 88.64 | .420 | -4.69 | 1.97 |
|  | Three Months – 12 Months | -0.13 | 89.92 | .899 | -3.82 | 3.36 |
|  | Six Months – 12 Months | 0.73 | 72.91 | .469 | -1.96 | 4.22 |
| HL | Three Months – Six Months | 0.23 | 76.56 | .816 | -3.62 | 4.58 |
|  | Three Months – 12 Months | -2.52 | 73.62 | **.014** | -8.83 | -1.03 |
|  | Six Months – 12 Months | -2.62 | 76.09 | **.011** | -9.52 | -1.30 |

*Note*: Bolded *p* values are statistically significant; EMM, Estimated Marginal Mean; NH, Normal Hearing; CI, Cochlear Implanted; HL, Unaided Hearing Loss.
